# U-shaped association between triglyceride-glucose index and acute kidney injury in critically ill children with cardiac diseases

**DOI:** 10.3389/fendo.2025.1598262

**Published:** 2025-09-25

**Authors:** Jiaxing Du, Keze Ma, Zhiwei Ye, Juanli Song, Sujun Chen, Zhenlin Xiong, Weinan Zheng, Meifei Li, Huiyi Yu, Fen Zhang

**Affiliations:** ^1^ Pediatric Intensive Care Unit, Dongguan Eighth People’s Hospital (Dongguan Children’s Hospital), Dongguan, Guangdong, China; ^2^ Nursing Department, Dongguan Eighth People’s Hospital (Dongguan Children’s Hospital), Dongguan, Guangdong, China; ^3^ Department of Surgery, Dongguan Eighth People’s Hospital (Dongguan Children’s Hospital), Dongguan, Guangdong, China; ^4^ Department of Neurology, Dongguan Eighth People’s Hospital (Dongguan Children’s Hospital), Dongguan, Guangdong, China

**Keywords:** TyG index, AKI incidence, children, critically ill, cardiac disease, insulin resistance, PIC database

## Abstract

**Background:**

The triglyceride–glucose (TyG) index, a surrogate of insulin resistance (IR), has been linked to acute kidney injury (AKI) in adults, but its relevance in critically ill children with cardiac disease is unclear. This study aimed to examine this relationship in this vulnerable population.

**Methods:**

This retrospective analysis drew on data from the Pediatric Intensive Care (PIC) database, comprising 2298 critically ill children treated in the Cardiac Intensive Care Unit (CICU) between 2010 and 2019. The primary outcome was the AKI incidence, whereas secondary outcome focused on the occurrence of severe AKI. Restricted cubic splines (RCS) assessed nonlinearity. Multivariate two-segment Cox regression analyses estimated hazard ratios (HRs) across TyG segments after adjustment for confounders. Subgroup analyses evaluated effect modification across clinical strata.

**Results:**

Among 2,298 CICU pediatric patients (48.7% male), 15.6% developed AKI. A U-shaped relationship between the TyG index and AKI was identified using RCS. Specifically, When the TyG index was below 8.602, an inverse relationship was observed with the primary outcome (HR 0.69, 95% CI 0.48–0.98). Conversely, values at or above 8.602 were positively associated with the AKI incidence (HR 1.63, 95% CI 1.15–2.31). Similarly, for the secondary outcome, the inflection was at TyG 8.757: <8.757, HR 0.53 (0.28–0.99); ≥8.757, HR 2.75 (1.18–6.42). Subgroup and sensitivity analyses reinforced the robustness of these findings.

**Conclusions:**

The TyG index showed a nonlinear, U-shaped association with AKI in critically ill children with cardiac disease, with inflection points around 8.602 (AKI) and 8.757 (severe AKI). These findings suggest that TyG may support bedside risk stratification in this population.

## Introduction

Treatment for critically ill children with cardiac diseases primarily involves medications, surgical interventions, and lifestyle modifications aimed at reducing symptoms and improving quality of life. However, In these patients, AKI often occurs and is significantly linked to an elevated risk of perioperative mortality (1). Reported AKI incidence after cardiac procedures ranges from 15% to 64% (2, 3). Moreover, AKI elevates perioperative mortality by 3 to 8 times (4, 5). AKI is also linked to long-term renal dysfunction, cardiac complications, and a reduced overall quality of life (6). These observations underscore the need for early identification and targeted interventions to improve outcomes.

Insulin resistance (IR) refers to impaired insulin sensitivity, resulting in increased circulating insulin levels and disrupting normal metabolic processes (7, 8). IR may impair kidney function via several pathways, including endothelial dysfunction, heightened inflammation, and tubulointerstitial fibrosis (9, 10). The TyG index, which reflects IR, is calculated by fasting triglycerides (TG) and fasting plasma glucose (FPG) (11, 12), has shown significant value in the context of AKI in recent years (13, 14). A substantial link between an increased TyG index and AKI has been observed in adult studies. Among individuals undergoing Coronary revascularization, the biomarker for contrast-induced AKI could be the TyG index. Across adult cohorts, including those undergoing coronary revascularization and those with traumatic brain injury, hypertension, or diabetes, the TyG index has been studied as an early indicator of AKI. (15–19). Across these adult cohorts, the TyG index is a practical tool for AKI risk assessment.

In pediatric populations, the TyG index correlates with insulin resistance and cardiometabolic risk (20, 21), underscoring its biological relevance in children. Because the TyG index integrates FPG and TG, it reflects dysglycemic and insulin-resistant states linked to renal vulnerability. Mechanistically, both hypoglycemia and hyperglycemia may precipitate AKI via sympathetic activation with hemodynamic instability, oxidative stress, endothelial dysfunction, and direct tubular injury (22, 23). Consistent with this biology, a U-shaped association between TyG index and short-term mortality has been reported in critically ill children (18). Accordingly, we hypothesized that the association between the TyG index and AKI in critically ill children with cardiac diseases may likewise be U-shaped.

To date, pediatric studies have used the TyG index primarily as a surrogate for insulin resistance and broader cardiometabolic risk, rather than as a marker of renal injury. Against this background, we examined the association between TyG and AKI in critically ill children with cardiac diseases. Given the high incidence of AKI in this population and its adverse long-term consequences, we further evaluated whether TyG could serve as an early, readily available biomarker for AKI risk to inform timely monitoring and management.

## Methods

### Data source and selection

The study is based on data gathered from the PIC database, specifically from children admitted to CICU (24, 25). The database includes 12,881 pediatric admissions from 2010–2019, of which 2,803 were CICU admissions, with detailed information on demographics, hospitalization, diagnoses, treatments, and outcomes. The data in the PIC database have undergone de-identification and do not involve personal privacy information. The usage of this data aligns with the Data Use Agreement (DUA). As a result, informed consent and ethical approval are not required for this study.

**Figure 1:**
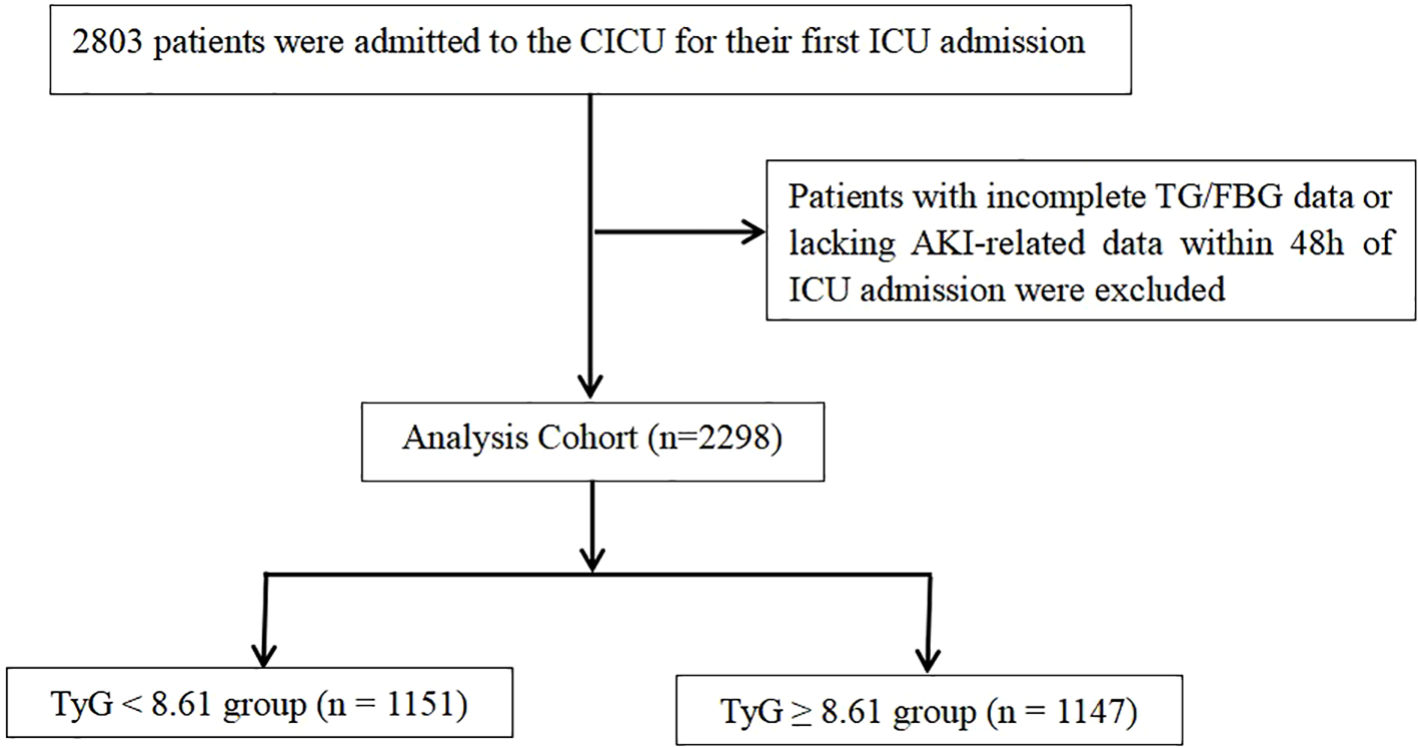
Diagram of the study population selection process.

This analysis includes data from 2,803 children admitted to the CICU, ranging from 1 month to 18 years of age. To minimize bias, only the first ICU admission was retained per patient. To ensure data integrity, we used a complete-case approach: records with missing analysis-specific variables and admissions lacking AKI data within the first 48 hours were excluded. No imputation was performed. The final cohort consisted of 2,298 patients, as depicted in **Figure 1**.

We extracted key variables from the PIC database using MySQL (version 8.0.39), included patient demographics, clinical laboratory indicators, and treatment interventions. Patient-related data were obtained during the initial 24 hours following ICU admission. For clinical laboratory indicators, we retained the first measurement recorded within that window. Demographic variables comprised age and gender. Clinical laboratory tests included albumin, blood urea nitrogen (BUN), cystatin-C, hemoglobin, total cholesterol, lymphocyte, neutrophil, activated partial thromboplastin time (APTT), platelet, white blood cell count (WBC), sodium, potassium, partial pressures of arterial oxygen (PaO_2_) and carbon dioxide (PaCO_2_). Treatment and intervention variables encompassed the use of vasopressors, insulin therapy, and surgical procedures, including atrial septal defect (ASD) repair, patent ductus arteriosus (PDA) repair, and ventricular septal defect (VSD) repair.

### Exposure and endpoints

To derive the TyG index, the following equation was applied: ln [TG (mg/dL) × FPG (mg/dL)/2] (26).This study focuses on AKI incidence as the primary outcome, characterized by a rise in serum creatinine (SCr) surpassing the pediatric reference change value optimized for AKI in children (pROCK) (27): the greater of an absolute increase of 20 μmol/L and a 30% rise from baseline in SCr within 7 days. The secondary outcome was severe AKI, defined as pROCK stage 2 or 3 (28): stage 2 was defined as an SCr increase of ≥40 μmol/L and ≥60%, and stage 3 as ≥80 μmol/L and ≥120% within 7 days. The lowest SCr value measured during the 7 days prior to admission is regarded the baseline level (29), if unavailable, the admission SCr was used. Outcomes were ascertained until hospital discharge or death, whichever occurred first.

### Statistical analysis

Depending on distribution, continuous variables are expressed as the mean ± SD or the median with IQR. The Student t-test is used for normally distributed data, whereas the Mann–Whitney U test is applied to non-normal distributions in group comparisons. Frequencies and proportions (%) describe categorical variables, and their comparisons rely on Fisher’s exact test or the Pearson chi-squared test.

The relationship between TyG index and AKI was analyzed using Cox proportional hazards regression models with multiple variables. Results were presented as hazard ratios (HR) with 95% confidence intervals (CI). Confounding was managed at different levels using three models: Model 1, unadjusted; Model 2, adjusted for demographics (age, gender) and therapeutic interventions (vasopressor use, insulin therapy, and cardiac surgical procedures: ASD, PDA, and VSD repairs); Model 3 underwent a comprehensive adjustment, including the adjustments from model 2 and additionally considering clinical laboratory tests, including albumin, BUN, cystatin-C, hemoglobin, total cholesterol, lymphocyte, neutrophil, APTT, platelet, WBC, sodium, potassium, PaO_2_, PaCO_2_. To assess nonlinearity, we fit an RCS Cox model using the Model 3 covariate set. As there is no established universal standard for the TyG index, the median value identified in this study was taken as the reference (30). The optimal TyG threshold was identified from the spline, and two-segment Cox regression models were then used to estimate associations below and above this threshold.

### Subgroup analyses

Subgroup analyses aimed to explore the potential effects of various population traits on the study outcomes. The analyses were stratified by key variables, including age (1 month–1 year, 1–5 years, and over 5 years), gender (male/female), use of vasopressors (yes/no), insulin therapy (yes/no), and surgical interventions (yes/no). To clarify treatment interactions, we fit Model 3 with multiplicative TyG×vasopressor and TyG×insulin interaction terms.

### Sensitivity analyses

To enhance robustness and mitigate confounding, we performed three sensitivity analyses. (1) We excluded children with severe condition (sepsis, shock, or malignancy) to reduce disease-severity bias. (2) We excluded ICU stays <12 hours to minimize bias from brief observation, rapid transfer, or early death. (3) We excluded surgical duration >6 hours as a pragmatic proxy for highly complex procedures, which often involve prolonged cardiopulmonary bypass, deep hypothermia, or a large transfusion burden; these factors may independently elevate AKI risk.

### Additional analyses

E-value analysis is employed to assess the potential impact of unmeasured confounding on our findings. E-values quantify the minimum strength of association that an unmeasured confounder would need with both TyG and AKI to explain away the observed association; larger E-values indicate greater robustness (31, 32). In this study, E-value analysis assessed the stability of the link between the TyG index and AKI incidence and to investigate whether unmeasured confounding factors could fully explain this association.

## Results

### Baseline characteristics

The study classified 2298 CICU children into two categories using the median TyG index: ≥ 8.61 (n=1147) and < 8.61 (n=1151) (**Table 1**). The central tendency of the age in the cohort was 15[6,41] months, with 48.7% male patients. Overall, 44.9% received vasopressor therapy and 25.8% received insulin therapy. Among the cardiac structural repair procedures, ASD repair accounted for 33.6%, PDA repair for 9.7%, and VSD repair was the most prevalent, comprising 43.6%. Compared with the TyG <8.61 group, children with TyG ≥8.61 were younger and had lower APTT, BUN, and neutrophil counts, and were less likely to receive vasopressor therapy. **Supplementary Table 1** displays the baseline characteristics categorized by the median TyG index and AKI.

**Table 1:** Study population characteristics classified by median TyG index.

Characteristics	Tyg index			
	Overall (N = 2298)	< 8.61 (N = 1151)	≥8.61 (N = 1147)	P value^a^
TyG	8.61[8.19,9.03]	8.20[7.89,8.41]	9.03[8.83,9.31]	<0.0001
Age, months	15[6,41]	20[8,49]	12[5,31]	<0.0001
Age category, (%)				<0.0001
1month-1year	963(41.9)	393(34.1)	570(49.7)	
1year-5years	960(41.8)	526(45.7)	434(37.8)	
≥5years	375(16.3)	232(20.2)	143(12.5)	
Gender, (%)				1
Female	1180(51.3)	587(51.0)	593(51.7)	
Male	1118(48.7)	564(49.0)	554(48.3)	
Clinical laboratory tests				
Albumin, g/L	44.2[41.6,46.4]	43.8[41,46.1]	44.5[42.2,46.6]	<0.0001
BUN, mmol/L	3.82[2.78,4.82]	3.95 [2.99, 4.88]	3.67 [2.48, 4.77]	0.0021
Cystatin-C, mg/dL	0.92[0.79,1.13]	0.88 [0.76, 1.05]	0.98 [0.83, 1.20]	<0.0001
Hemoglobin, g/L	121[112,129]	121[112,129]	121 [112,129]	1
Total cholesterol, mmol/L	3.7[3.2,4.3]	3.6[3.0,4.2]	3.9[3.3,4.4]	<0.0001
Lymphocyte, %	57.8[46.0,66.5]	54.7[42,64.3]	60.3 [50.7,68.3]	<0.0001
Neutrophil, %	32.2[23.68,44.1]	35.4[25.9,48.2]	29.4 [22.0,39.0]	<0.0001
Aptt, seconds	27.4[26.5,28.7]	27.6[26.5,28.7]	27.1[26.4,28.7]	1
Platelet, 109/L	329[270,400]	321[262,391]	339[279,409]	<0.0001
WBC,109/L	8.76[7.08,10.86]	8.5[6.9,10.5]	9.1[7.4,11.2]	<0.0001
Sodium, mmol/L	135[133,138]	135[134,138]	135[133,138]	0.5091
Potassium, mmol/L	3.6[3.4,3.9]	3.6[3.4,3.8]	3.6 [3.4,3.9]	1
PaO2, mmHg	184[145,210]	183[150,211]	184[143,209]	1
PaCO2, mmHg	35.8[32.8,39.7]	35.5[32.6,39.3]	36.2[33.1,40.2]	0.0017
Use of vasopressors, (%)				0.0042
Yes	1031(44.9)	561(48.74)	470(41.0)	
No	1267(55.1)	590(51.26)	677(59.0)	
Insulin therapy, (%)				0.1512
Yes	592(25.8)	325(28.24)	267(23.3)	
No	1706(74.2)	826(71.76)	880(76.7)	
ASD repair, (%)				1
Yes	772(33.6)	390(33.88)	382(33.3)	
No	1526(66.4)	761(66.12)	765(66.7)	
PDA repair, (%)				1
Yes	222(9.7)	103(8.9)	119(10.4)	
No	2076(90.3)	1048(91.1)	1028(89.6)	
VSD repair, (%)				1
Yes	1002(43.6)	494(42.9)	508(44.3)	
No	1296(56.4)	657(57.1)	639(55.7)	

TyG, triglyceride-glucose; BUN, blood urea nitrogen; APTT, activated partial thromboplastin time; WBC, white blood cell count; PaO2, arterial oxygen pressure; PaCO2, carbon dioxide pressure; ASD, atrial septal defect; PDA, patent ductus arteriosus; VSD, ventricular septal defect.

a P values are adjusted for multiple comparisons across all baseline variables using the Bonferroni method (m = 23).

### Association between TyG index and AKI

**Figure 2:**
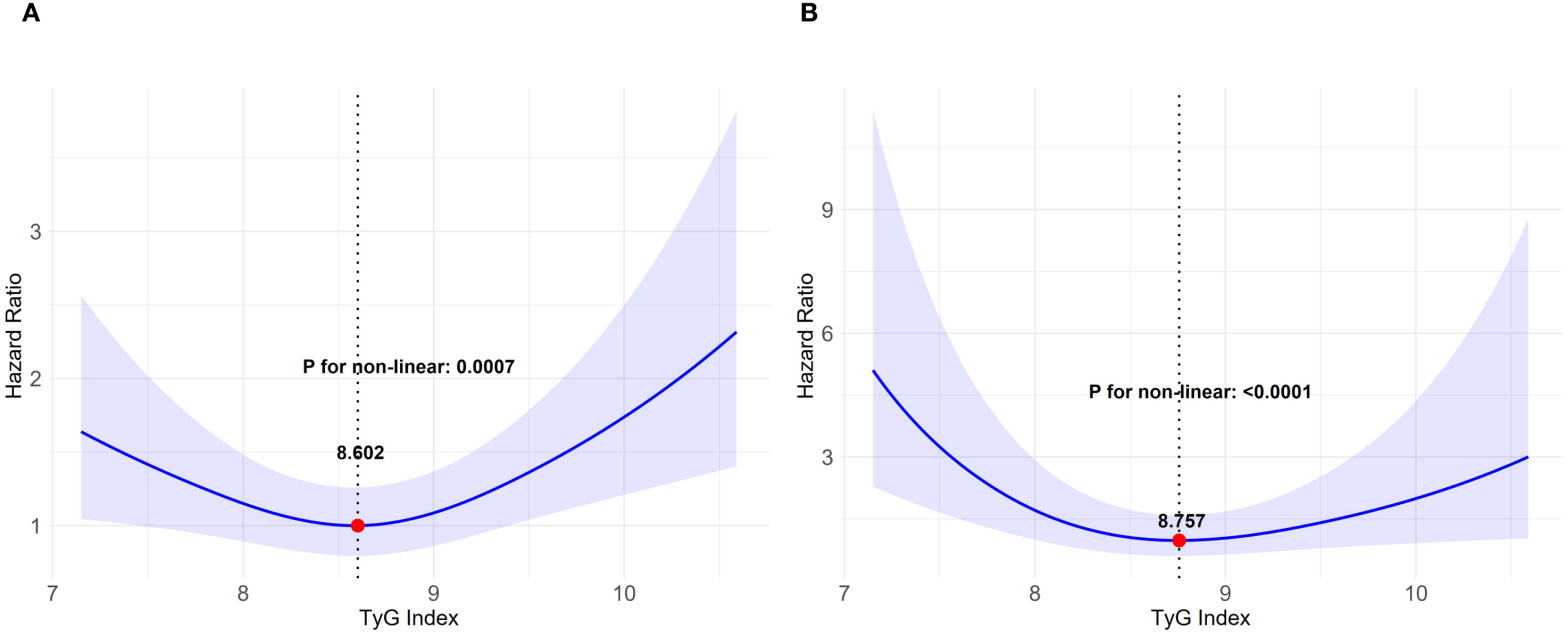
Relationship between the TyG index and AKI depicted by restricted cubic spline curves. TyG triglyceride-glucose, AKI acute kidney injury. **(A)** AKI incidence, **(B)** occurrence of severe AKI. The model underwent a comprehensive adjustment, including demographic characteristics, therapeutic interventions and clinical laboratory tests.

The AKI incidence among the study cohort was 15.6% (358 cases), while that of severe AKI was 3.3% (76 cases). Following the adjustment for demographic factors, therapeutic interventions, and clinical laboratory tests, the RCS analysis revealed a U-shaped association between the TyG index and both the primary and secondary outcomes, with inflection points of 8.602 and 8.757, and both P values for the non-linear relationship < 0.001 (**Figure 2**). We applied a multivariable, two-segment Cox regression model for further analysis. For TyG index lower than 8.602, a one-unit increase resulted in a 41% drop in AKI incidence (HR 0.69, 95% CI 0.48–0.98). With a TyG index exceeded 8.602, the AKI incidence increased significantly by 63% (HR, 1.63, 95% CI 1.15–2.31). In the secondary outcome analysis, further findings showed that below 8.757, the occurrence of severe AKI notably declined as the TyG index increased. (HR 0.53, 95% CI 0.28–0.99). Conversely, for TyG indices at least 8.757, higher values were linked to a significant rise in the occurrence of severe AKI. (HR 2.75, 95% CI 1.18–6.42). For a detailed overview of the results, refer to **Table 2**.

**Table 2:** Association between TyG index and outcomes in critically ill children with cardiac diseases.

Tyg index (per unit increase)	Total, N	No. of events (incident rate, %)	Model 1		Model 2		Model 3	
			HR [95% CI]	P value	HR [95% CI]	P value	HR [95% CI]	P value
Primary outcome: AKI incidence								
< 8.602	1135	161	0.68[0.49,0.95]	0.0222	0.70[0.52,0.95]	0.0240	0.69[0.48,0.98]	0.0394
≥ 8.602	1163	197	1.6[1.19,2.13]	0.0016	1.49[1.11,1.99]	0.0075	1.63[1.15,2.31]	0.0061
Secondary outcome: occurrence of severe AKI								
< 8.757	1345	188	0.38[0.29,0.51]	<0.0001	0.46[0.35,0.60]	<0.0001	0.53[0.28,0.99]	0.0476
≥ 8.757	953	170	3.39[1.72,6.72]	0.0005	3.61[3.61,7.18]	0.0003	2.75[1.18,6.42]	0.0193

### Subgroup analyses

**Figure 3:**
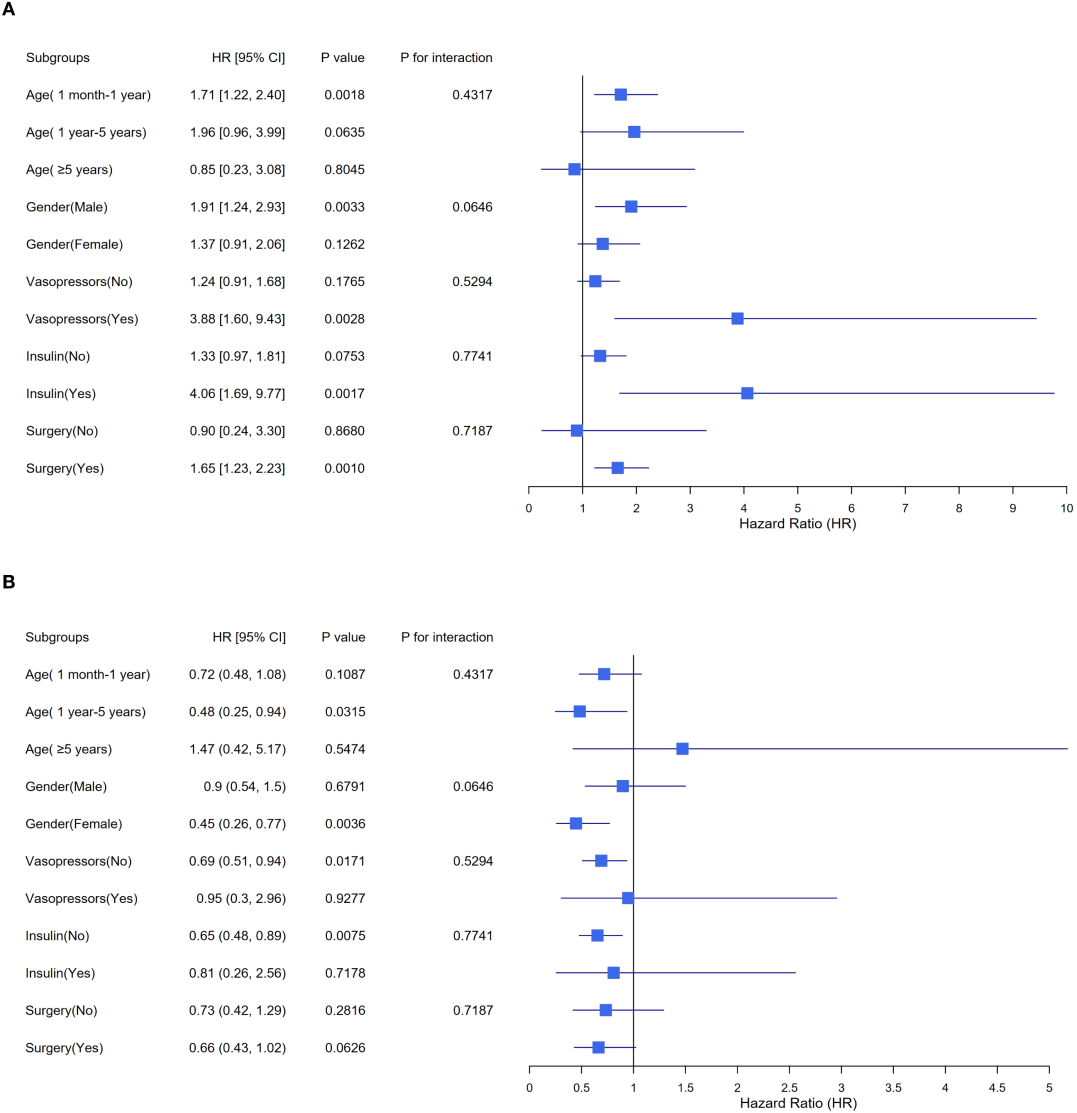
Stratified analyses exploring the impact of potential factors between TyG index and AKI incidence. TyG triglyceride-glucose, AKI acute kidney injury, HR hazard ratio, CI confidence interval. **(A)** TyG index < 8.61, **(B)** TyG index ≥ 8.61. The model underwent a comprehensive adjustment, including demographic characteristics, therapeutic interventions and clinical laboratory tests.

**Figure 3** illustrates the findings from the subgroup analyses examining the association between the TyG index and AKI incidence. The consistency of this relationship was observed in various subgroups classified by age, gender, and surgery. By contrast, treatment interactions varied. Among children with TyG ≥ 8.61, the adjusted HR was 1.24 (95% CI 0.91–1.68) without vasopressor therapy versus 3.88 (1.60–9.43) with vasopressor therapy. For insulin, the corresponding HRs were 1.33 (0.97–1.81) without versus 4.06 (1.69–9.77) with therapy. These results indicate that, among individuals with elevated TyG indices, vasopressor use and insulin therapy were associated with higher adjusted AKI risk, with wide CIs reflecting small treated subgroups. This pattern is further illustrated in **Supplementary Figure 1**.

### Sensitivity analyses

Sensitivity analyses (**Supplementary Table 2**) showed modest shifts in HRs under progressively stricter exclusions. For TyG < 8.602, the adjusted HR moved slightly downward from 0.69 (95% CI 0.48–0.98) in the main analysis to 0.65 (0.43–0.98) after excluding severe condition (analysis 1); 0.64 (0.43–0.97) after additionally excluding ICU stays < 12 hours (analysis 2), and 0.62 (0.41–0.94) after also excluding surgical duration > 6 hours (analysis 3). For TyG ≥ 8.602, estimates were stable: 1.63 (1.15–2.31) in the main analysis, 1.61 (1.18–2.20) in analyses 1 and 2, and 1.65 (1.20–2.26) in analysis 3. Confidence intervals were similar or slightly narrower under stricter criteria, and all associations remained statistically significant (P < 0.05).

### Additional analyses

Using the TyG strata <8.601 and ≥8.601, the E-values for the association with AKI were 2.26 and 2.24, respectively. Within model 3, the highest identified HRs among confounding factors were insulin (HR 1.79) and APTT (HR 1.40). Since unmeasured confounders would require an adjusted HR above 2.26 to account for the observed link between the TyG index and AKI incidence, residual confounding alone is unlikely to explain this relationship.

## Discussion

To our knowledge, this large retrospective study is the first to examine the association between the TyG index and AKI in children with cardiac disease. After adjustment for confounders, we observed a nonlinear, U-shaped association with both AKI and severe AKI. Spline analyses identified inflection points at 8.602 and 8.757, respectively. These findings highlight dual clinical implications: the TyG index may serve as an early, readily available marker to support AKI risk stratification in practice. Additionally, for dynamic bedside monitoring, given the small difference between the two cut-points, we prefer to interpret these values as evidence for a shared low-risk band (8.60–8.76) rather than as rigid single-number cut-offs. This difference is likely driven by several considerations. Clinically, severe AKI typically requires a stronger metabolic and hemodynamic insult to become manifest; therefore, at higher TyG levels the point at which risk begins to rise is expected to be slightly right-shifted relative to AKI. In our cohort, event frequency also differed markedly (AKI 15.6% vs severe AKI 3.3%). With fewer events, the spline-based change-point is more susceptible to sampling variability and tends to gravitate toward regions with denser observations, yielding a modestly higher threshold.

Because the TyG index jointly reflects dysglycemia and triglyceride-related lipotoxic stress and serves as a validated surrogate of insulin resistance (33), it may reflect biology relevant to AKI more completely than fasting glucose or triglycerides alone. Clinically, this translates into pragmatic benefits at the bedside: the TyG index is derived from FPG and TG, can be auto-calculated for real-time risk flagging, and requires no additional assays, phlebotomy, or turnaround time. The observed U-shaped pattern with a narrow low-risk band further supports avoiding both hypoglycemia and marked hyperglycemia and using alerts based on TyG to prompt early monitoring and metabolic care. In contrast, injury or functional biomarkers such as neutrophil gelatinase-associated lipocalin (NGAL) and cystatin C primarily reflect tubular injury or filtration impairment and therefore require appropriate post-injury timing; by comparison, the TyG index functions as an antecedent risk marker obtainable before overt damage (34, 35). Accordingly, the TyG index should be viewed as complementary, not a replacement, to organ-specific injury markers.

Over the past few years, the TyG index has attracted growing interest as a potential indicator of AKI risk. However, research on this association remains limited, with most studies focusing on adult populations. Multiple studies report a positive association between TyG and AKI across diverse conditions. Among patients undergoing coronary revascularization, The highest TyG quartile (Q4) showed an 89% increased risk of AKI relative to the lowest quartile (Q1) (HR 1.89, 95% CI 1.12–3.17, P = 0.017) (33). Similarly, in patients with heart failure, A one-unit boost in the TyG index correlated with a 58% higher risk of AKI (HR 1.58, 95% CI 1.22–2.04, P = 0.0006) (36). In patients with hypertension, A TyG index ≥4.803 substantially heightened AKI risk (P < 0.001), the top quartile had a 66% incidence, significantly exceeding than the 47% seen in the lowest group (P < 0.001) (15). In sepsis, A rise in the TyG index corresponded to a greater risk of developing AKI (HR 1.073, 95% CI 1.005–1.147, P = 0.036) (37). Collectively, higher TyG is associated with greater AKI risk across adult cohorts; however, its relationship with AKI in children with cardiac disease has not been defined. The present study helps to fill this gap. and provides foundational data for future research.

In contrast to previous studies, we observed a U-shaped association between the TyG index and AKI in children with cardiac disease, rather than the positive linear association described previously. A primary explanation is endpoint definition: compared with adult Kidney Disease: Improving Global Outcomes (KDIGO) criteria (38), the pediatric pROCK uses lower diagnostic thresholds and is easier to trigger (27), yielding milder AKI; such definition differences alter case ascertainment and the severity mix, potentially changing the shape of the association. A second contributor is exposure distribution. Our cohort’s median TyG was 8.61, whereas adult cohorts typically centered higher (about 9.15), so prior analyses likely operated in a range where risk appears more monotonic. Consistent with a nonlinear framework, three studies of atrial fibrillation, all-cause mortality, and incident diabetes reported lowest risk at intermediate TyG, with higher risks at both tails (18, 39, 40); their TyG ranges were similar to or lower than ours. Taken together, differences in AKI definitions and TyG distributions across settings likely underlie the discrepant association shapes.

We propose several mechanisms that may underlie the U-shaped relationship between the TyG index and AKI. The TyG index is a composite measure derived from TG and FPG, has been proven to be a reliable indicator for evaluating IR (41). When the TyG index exceeds 8.602, higher TyG may be associated with greater AKI risk through IR-related pathways, including: (1) induction of glomerular hyperfiltration, where abnormal hemodynamic changes may aggravate tubular dysfunction (42); (2) activation of the sympathetic nervous system and the renin-angiotensin-aldosterone system (RAAS), which, when excessively activated, not only induces vasoconstriction and reduces renal blood flow but also increases renal pressure, further impairing renal perfusion and function (43, 44); (3) oxidative stress triggered by IR, resulting in excessive production of reactive oxygen species (ROS), which damage renal cells, particularly mitochondria, resulting in cellular dysfunction (45, 46); and (4) IR-induced inflammatory responses that promote the release of inflammatory cells and cytokines, creating a chronic inflammatory environment that accelerates renal deterioration (47). When TyG < 8.602, the inverse association may be associated with a hypoglycemic metabolic state. The mathematical composition of the TyG index suggests that low FPG (<4.0 mmol/L) is a likely driver of this pattern. Systematic reviews indicate that decreased FPG contributes to both a higher incidence of all-cause mortality and an increased likelihood of cardiac events (48). Furthermore, studies examining different metabolic patterns have confirmed a significant association between AKI and the low glucose/high lactate metabolism pattern (49). We speculate that hypoglycemia may impair insulin sensitivity and energy metabolism (50, 51), ultimately adversely affecting renal function and contributing to the development of AKI.

For subgroups with a TyG index ≥ 8.602, the within-subgroup association between TyG and AKI risk was attenuated without vasopressor or insulin therapy (HR 1.24, 0.91–1.68; HR 1.33, 0.97–1.81, respectively) but was markedly steeper among those receiving vasopressor therapy (HR 3.88, 1.60–9.43) or insulin therapy (HR 4.06, 1.69–9.77). This discrepancy may be attributed to the differing pathophysiological states of the two groups. Patients not receiving vasopressors typically exhibit better circulatory stability, suggesting less severe underlying disease and relatively intact compensatory hemodynamic mechanisms, which may mitigate the metabolic damage to the kidneys associated with an elevated TyG index. Additionally, patients receiving insulin therapy inherently display more pronounced metabolic disturbances, particularly in the context of poor FPG control. IR and inadequate FPG control may lead to a series of metabolic issues that can increase renal burden, thereby escalating the AKI incidence. However, these treatment-stratified findings should be interpreted with caution given the small number of events and wide confidence intervals in treated subgroups and the potential for confounding by indication.

This study, utilizing a long-term cohort dataset from PIC database (2010–2019, n=2298), strengthens the reliability of its findings through a large sample size and multidimensional sensitivity analyses, including an E-value analysis. The decade-long time frame may aid generalizability; nevertheless, several limitations warrant caution. First, due to the study’s retrospective and observational design, it cannot confirm clear causal links, although it identifies the relationship between the TyG index and AKI. Second, relying on data from a single research facility may introduce selection bias, limiting the applicability of the conclusions to populations from other institutions or adult patients. Third, despite E-value analyses, residual confounding may persist and could affect effect estimates. Fourth, the precision of the severe AKI estimate was limited (HR 2.75; 95% CI 1.18–6.42), due to the lower event count. therefore, this finding should be interpreted with caution. Finally, the absence of longitudinal data on long-term renal outcomes precluded evaluating the TyG index’s prognostic value for chronic kidney dysfunction. These limitations highlight the need for multicenter prospective cohorts with serial metabolomic profiling and extended follow-up to clarify mechanisms linking the TyG index to AKI.

## Conclusion

This study shows a nonlinear, U-shaped association between the TyG index and AKI in children with cardiac disease, with an inflection point at 8.602. This finding supports the potential use of TyG for bedside risk stratification in pediatric cardiac care and may provide a simple, actionable indicator to identify children at increased risk of AKI.

## Data Availability

The raw data supporting the conclusions of this article will be made available by the authors, without undue reservation.

## Glossary

TyG: triglyceride-glucose
IR: insulin resistance
AKI: acute kidney injury
PIC: Pediatric Intensive Care
CICU: Cardiac Intensive Care Unit
RCS: restricted cubic splines
TG: triglycerides
FPG: fasting plasma glucose
DUA: Data Use Agreement
BUN: blood urea nitrogen
APTT: activated partial thromboplastin time
WBC: white blood cell count
PaO_2_: partial pressures of arterial oxygen
PaCO_2_: partial pressures of carbon dioxide
ASD: atrial septal defect
PDA: patent ductus arteriosus
VSD: ventricular septal defect
SCr: serum creatinine
pROCK: pediatric reference change value optimized for AKI in children
HR: hazard ratios
CI: confidence intervals
RAAS: renin-angiotensin-aldosterone system
ROS: reactive oxygen species
NGAL: neutrophil gelatinase-associated lipocalin
KDIGO: Kidney Disease: Improving Global Outcomes.
