# Supplementary figures and images for: U-shaped association between triglyceride-glucose index and acute kidney injury in critically ill children with cardiac diseases
Source: Front Endocrinol (Lausanne). 2025 Sep 25;16:1598262. doi: 10.3389/fendo.2025.1598262 (PMC12507589; doi:10.3389/fendo.2025.1598262)

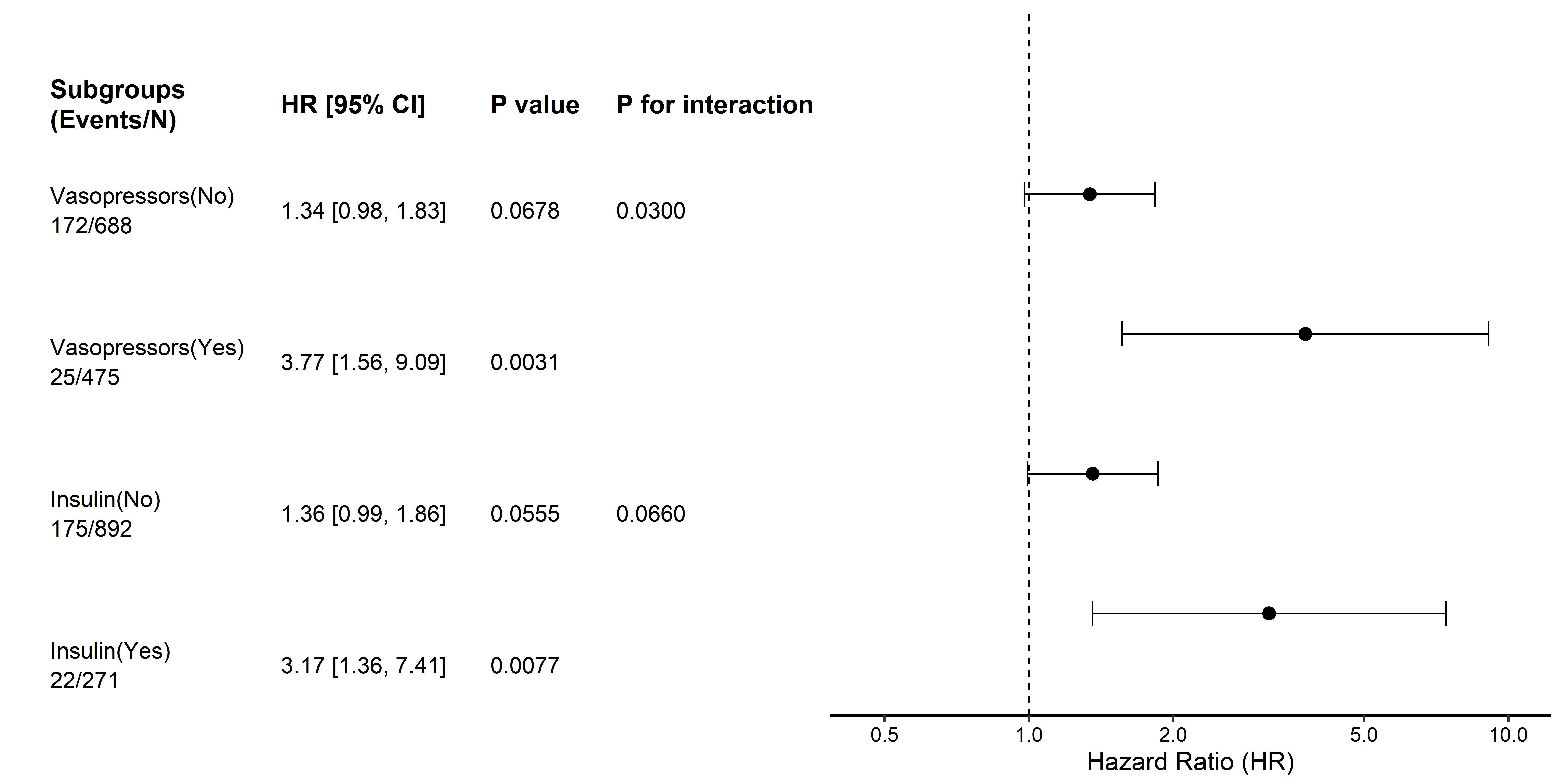

Supplement: Supplementary file 1 [file Image1.jpeg]
